# Enhancing COVID 19 vaccination coverage in Sierra Leone

**DOI:** 10.1093/eurpub/ckac131.019

**Published:** 2022-10-25

**Authors:** R Moretti, T Scognamiglio, A Zoli, A Miozzo, P Lansana, A Nunie

**Affiliations:** 1 Primary Health Care, ATS Bergamo, Bergamo, Italy; 2 Direction, AREU, Milan, Italy; 3 Freetown Urban Health District, EPI District, Freetown, Sierra Leone; 4 Direction, EPI National, Freetown, Sierra Leone

## Abstract

The project aim to support Sierra Leone in enhancing vaccination coverage. BACKGROUND In November 2021, the vaccination coverage rate was 7% for one dose and 4% for two. According with EPI program, the main problem was not the lack of vaccines (already provided by different donors) but the need of support (training, motivation, allowances) to health personnel. Lombardy project involved 31 local vaccination teams performing a refresh training, followed, by micro-planning meetings. SIGNIFICANCE The Covid epidemiology in SL is not well known, but the possible new waves makes this intervention a high public health priority. USEFULNESS. The model is based on training, motivation, follow up and a very simple monetary allowance system based on performance. An online daily report available on smartphone was provided to follow up performances and manage allowances system. PROBLEM The objective was a donation of vaccines, but after a short assessment the issue changed to the need of supporting the local health staff. The goal shifted to train, give incentives for health workers, support to micro-planification and also to work on timely reporting to follow up the project results. QUESTIONS ANSWERED. Can support to microplanning be effective in enhancing vaccination numbers? Can monetary incentives to personnel based on performances enhance vaccination numbers? Can a setting approach (school, workplaces,..) enhance vaccinations numbers? RESULTS the numbers of vaccination Increased from an average of 5 per team by day in early December (after the refresh training) up to an average of 15 by day after the support to micro-planning, the monetary progressive incentives based on performance and the introduction of settings approach. Up to 85.000 doses was performed in 4 months.

**Key messages:**

• Planning, performance based payement and training can enhance vaccination coverage.

• African countries can performe good vaccination programs if supported in organization and non only in vaccine donation.

